# Comprehensive Genome Analysis of Colistin-Only–Sensitive KPC-2 and NDM1-1-Coproducing *Klebsiella pneumoniae* ST11 and *Acinetobacter baumannii* ST2 From a Critically Ill Patient With COVID-19 in Saudi Arabia: Whole Genome Sequencing (WGS) of *K. pneumoniae* ST11 and *A. baumannii* ST2

**DOI:** 10.1155/2024/9233075

**Published:** 2024-10-29

**Authors:** Ibrahim A. Al-Zahrani, Thamer M. Brek

**Affiliations:** ^1^Medical Laboratory Sciences Department, Faculty of Applied Medical Sciences, King Abdulaziz University, Jeddah, Saudi Arabia; ^2^Special Infectious Agents Unit-Biosafety Level-3, King Fahad Medical Research Centre, King Abdulaziz University, Jeddah, Saudi Arabia; ^3^Public Health Laboratory, The Regional Laboratory, Jazan Health Cluster, Jazan, Saudi Arabia

**Keywords:** *A. baumannii* ST2, carbapenem-resistant, COVID-19, *K. pneumoniae* ST11, NDM and KPC, Saudi Arabia

## Abstract

The COVID-19 pandemic has intensified the issue of multidrug-resistant (MDR) infections, particularly in intensive care units (ICUs). This study documents the first known case of coinfection with two extensively drug-resistant (XDR) bacterial isolates in a critically ill patient with COVID-19 in Saudi Arabia. Both XDR isolates were recovered from blood and were resistant to all tested antimicrobial agents except colistin. Whole genome sequencing (WGS) revealed that the *K. pneumoniae* isolate KP-JZ107 had sequence type 11 (ST11) and core genome MLST (cgMLST 304742), while the *A. baumannii* isolate AB-JZ67 had ST2 and cgMLST 785. KP-JZ107 was found to possess the virulence plasmid KpVP-type-1, carbapenemase genes *bla*_*NDM*_ and *bla*_*KPC*_, and numerous antimicrobial-resistant genes (ARGs). The AB-JZ67 isolate had several biofilm-related genes, including biofilm-associated protein (BAP), csuE, and pgaB, and multiple ARGs, including *bla*_*ADC*−25_, *bla*_*OXA*−23_, and *bla*_*OXA*−66_. Our findings suggest that the coexistence of KP-JZ107 and AB-JZ67 isolates may indicate their widespread presence in ICUs, requiring comprehensive surveillance studies across all hospitals.

## 1. Introduction

Multidrug-resistant (MDR) bacteria have posed a serious threat to public health in recent years. The emergence and spread of these pathogens have been aggravated by the COVID-19 pandemic, which has further highlighted the need for effective infection control measures [1]. Some individuals diagnosed with COVID-19 may require admission to the intensive care unit (ICU) to manage severe symptoms of the disease. However, admission to the ICU increases the likelihood of infection with MDR bacteria, particularly carbapenemase-producing *Klebsiella pneumoniae* (CPKP) and *Acinetobacter baumannii*. These Gram-negative bacteria typically inhabit the respiratory and gastrointestinal systems and can lead to various infections such as pneumonia, urinary tract infections, and bloodstream infections (BSIs) [2].

*K. pneumoniae* strains have several virulence factors that enable them to overcome the host's immune responses, including capsule, lipopolysaccharides, fimbriae, iron carriers, and efflux pumps [3]. The polysaccharide capsule, for example, plays a role in protecting bacteria from phagocytosis by macrophages and bactericidal factors. Bacterial fimbriae are also major virulence factors that attach to host cells and facilitate infection. In addition, *K. pneumoniae* has iron-acquiring systems called siderophores, which are secreted molecules that play an important role in bacterial growth and reproduction within host cells [4]. *A. baumannii* has an arsenal of virulence factors that enable it to cause infection and contribute to prolonged colonization of the host and hospital environment. These factors include outer membrane protein A (ompA), lipopolysaccharide, capsule, phospholipase, secretion systems (I, II, V, and IV), biofilm formation, efflux pumps, outer membrane vesicles, and acquisition of nutrient (iron, zinc, and magnesium) [5].

There are many risk factors associated with developing a serious infection caused by *K. pneumoniae* and *A. baumannii*, which include prolonged hospitalizations, immunodeficiency diseases, old age, chronic disease, antimicrobial use, and exposure to invasive procedures, such as catheters or mechanical ventilation. Moreover, gastrointestinal colonization by both organisms in ICU patients is a risk factor for the acquisition of antimicrobial resistance (AMR) and constitutes a major source of nosocomial infections [6]. Patients who are critically ill due to COVID-19 face a heightened risk of developing secondary bacterial infections, such as ventilator-associated pneumonia (VAP) and BSIs. These infections are often caused by MDR organisms and can be particularly challenging to manage within the ICUs. Thus, there is an urgent need to develop effective strategies for early detection and management of these infections to prevent their spread and reduce the burden on healthcare systems. In the current study, isolates were recovered from a critical ill patient with COVID-19 and septicemia infection in a tertiary hospital, Jazan, Saudi Arabia. Furthermore, we performed a genomic analysis of two extensively drug-resistant (XDR) isolates *K. pneumoniae* KP-JZ107 and *A. baumannii* AB-JZ67.

## 2. Methods

### 2.1. Bacterial Identification and Antibiotic Susceptibility Tests

During routine testing in microbiology laboratories, two clinical XDR isolates *K. pneumoniae* and *A. baumannii* were recovered from the blood of a critically ill patient with COVID-19 in the ICU of a tertiary hospital, Jazan, Saudi Arabia. One of the isolates, KP-JZ107, was among a collection of carbapenem-resistant isolates that were published by Brek et al. [7]. Identification and antimicrobial susceptibility testing (AST) were performed using a BD phoenix M50 instrument (Becton Dickinson, USA). The AST panel included carbapenems (imipenem, meropenem, and ertapenem), amoxicillin–clavulanate, amikacin, ampicillin, ceftazidime, cefepime, ciprofloxacin, gentamicin, piperacillin–tazobactam, tigecycline, and trimethoprim sulfamethoxazole. The AST results were interpreted in accordance with the M100-S28 guidelines of the Clinical and Laboratory Standards Institute (CLSI). Both isolates were transferred to 1.5-mL tubes containing Luria–Bertani (LB) broth and 20% (v/v) glycerol and stored at −80°C for further analysis.

### 2.2. Molecular Detection of Carbapenemase Genes

Genomic DNA from the bacterial isolates was suspended by using a loop full of pure bacterial culture into a microcentrifuge tube containing 300 μL of sterile deionized water. The suspension was heated at 95°C for 15 min using a heat block, followed by centrifugation at 13,000 g for 10 min. The supernatant (2 μL) was used as the DNA template for polymerase chain reaction (PCR). The presence of five common carbapenemase-encoding genes (*bla*_KPC_, *bla*_VIM_, *bla*_IMP_, *bla*_NDM_, and *bla*_OXA−48_) in the *K. pneumoniae* isolates was investigated using a multiplex PCR with primers previously described by Al-Zahrani and Alasiri. The primers were obtained from Macrogen (Seoul, South Korea). Each 50 μL PCR reaction contained 25 μL of Dream Taq PCR Master Mix (Thermo Fisher Scientific, USA), 13 μL of sterile, RNase-free water, 1 μL (1 μM final concentration) of each primer, and 2 μL of DNA template. Amplification steps were performed according to the protocol reported by Brek et al. [7, 8]. The presence of oxacillinase genes (*bla*_*OXA*−51_, *bla*_*OXA*−23_, *bla*_*OXA*−24_, and *bla*_*OXA*−58_) in the *A. baumannii* isolate was investigated by PCR using primers designed by Mostachio et al. [9]. The final PCR reaction mixture had a total volume of 50 μL and contained a concentration of 1 pmol/μL of each primer, along with the aforementioned reaction-mixture components. The PCR conditions comprised an initial cycle of 94°C for 5 min and 30 cycles at 94°C for 25 s, 52°C for 40 s, and 72°C for 50 s. A final cycle was performed at 72°C for 6 mins. The amplified PCR products were analyzed by using gel electrophoresis on 2% agarose gel and visualized using a gel imaging system (Bio-Rad Laboratories, USA). The resulting PCR products with more than one gene in a single isolate were confirmed by using singleplex PCR. The positive controls included the following strains: *K. pneumoniae* NCTC 13438 as a positive control for KPC, NCTC 13443 for NDM, NCTC 13440 for VIM, NCTC 13442 for OXA-48, and *E. coli* NCTC 13476 for IMP. *E. coli* NCTC 10418 was used as a negative control. *A. baumannii* ATCC® 19606 TM was used as a positive control for blaOXA-51.

### 2.3. DNA Extraction for Whole Genome Sequencing (WGS)

The bacterial isolates were grown in LB broth overnight at 37°C. Genomic DNA from harvested cultures was extracted using a GeneJET Genomic DNA Purification Kit (Thermo Scientific, Lithuania) according to the manufacturer's instructions. The yielded DNA sample was quantified using a NanoDropTM 1000 spectrophotometer (Thermo Scientific, MA, USA) and stored at −20°C.

### 2.4. WGS and Data Analysis

WGS was carried out as previously described by Yasir et al. [10] using an MiSeq system (Illumina Inc., San Diego, CA, USA) with v.1, 2 × 150-bp chemistry. A minimum depth coverage of 60× was achieved for both genomes. The genome assembly was prepared from high-quality filtered reads using the Unicycler v0.4.8. Genome annotation was carried out using the PATRIC web resources (https://www.patricbrc.org/). Antimicrobial-resistant genes (ARGs) and virulence genes were identified using ResFinder 4.1 and 4.6 and VirulenceFinder 2.0, respectively. The sequence type (ST) and core genome MLST (cgMLST) of the isolates were identified using the Center for Genomic Epidemiology web server (https://cge.food.dtu.dk/services/MLST/), while Kaptive software (Kaptive bio.tools) was used to determine the capsular serotype of our isolates. The CGView server (https://proksee.ca) was used to construct the genome maps. A phylogenetic tree was built using the PATRIC web resources https://www.bv-brc.org/app/PhylogeneticTree, and visualized by Interactive Tree of Life (iTOL) (https://itol.embl.de/). All raw sequencing data are available in the NCBI database (BioProject ID PRJNA110342).

## 3. Results

### 3.1. Clinical Information

A 69-year-old female patient was admitted to the ICU at a tertiary care hospital in the Jazan region due to a severe infection with acute respiratory syndrome coronavirus 2 (SARS-CoV-2), which led to acute respiratory dysfunction. During her hospital stay, she was also diagnosed with a secondary bacterial infection caused by *K. pneumoniae* and *A. baumannii*. Consequently, the patient developed septicemia, and both bacterial isolates were identified from her blood culture.

### 3.2. Antibiotic Susceptibilities

The AST results showed that both the *K. pneumoniae* and *A. baumannii* isolates were resistant to all tested antimicrobial agents except colistin, which suggests that both strains are XDR. Both strains showed the same noticeable resistance to imipenem (> 8 mg/L), meropenem (> 8 mg/L), and ertapenem (> 4 mg/L). The AST results of various antimicrobial agents are shown in Table 1.

### 3.3. Bacterial Isolates Harboring Carbapenemase Genes

We examined whether the two isolates contained genes associated with carbapenem resistance. To this end, multiplex-PCR assays were used to determine the presence of widespread carbapenemase genes (*bla*_KPC_, *bla*_VIM_, *bla*_IMP_, *bla*_NDM_, and *bla*_OXA−48_) in *K. pneumoniae* and the most frequent *bla*_*OXA*−*LIKE*_ genes (*bla*_OXA−51_, *bla*_OXA−23_, *bla*_OXA−24/40_, and *bla*_OXA−58_) in the *A. baumannii* isolate. In the *K. pneumoniae* isolate, both *bla*_NDM_ and *bla*_KPC_ were found, while the *bla*_OXA−LIKE_ genes *bla*_OXA−51_ and *bla*_OXA−23_ were detected in the *A. baumannii* isolate.

### 3.4. WGS

**Figure 1:**
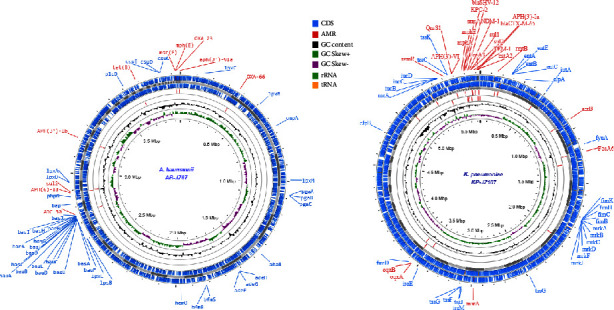
Genomic maps of *K. pneumoniae* ST11 (KP-JZ107) and *A. baumannii* ST2 (AB-JZ67) isolates. Coding sequences (CDSs) are denoted by blue lines, while contigs are indicated by gray arrows. The GC skew+ is represented by green peaks, GC skew- is represented by by purple, and G + C content is depicted by black peaks. The virulence genes are labeled blue and AMR genes red. The maps were constructed utilizing the CGView server (accessed at https://proksee.ca/ on December 20, 2023). AMR: antimicrobial resistance.

The RAST tool kit (RASTtk) was used to annotate the genome of *K. pneumoniae* KP-JZ107 with genetic code 11, which was assigned a unique genome identifier of 573.54566. The assembly of the KP-JZ107 genome produced 151 contigs and a draft genome size of 5,838,532 bp with a G + C content of 56.7%. The chromosome contained 5,933 predicted coding sequences (CDSs), 4 ribosomal RNA (rRNA) genes, and 76 transfer RNA (tRNA) genes (Figure 1).

The AB-JZ67 genome was assigned a unique genome identifier of 470.13565 and had 82 contigs with a total length of 3,950,589 bp and an average G + C content of 38.78%. This assembled genome had 3,818 CDSs, 63 tRNA genes, and 3 rRNA genes (Figure 1).

### 3.5. Identification of STs and Capsular Serotypes

Multilocus sequence typing (MLST) analysis using the Center for Genomic Epidemiology web server revealed that the KP-JZ107 isolate had ST11 and cgMLST 304742, while AB-JZ67 isolate had ST2 and cgMLST 785. According to the Kaptive software, the capsular serotype of KP-JZ107 and AB-JZ67 was identified as capsule locus KL47 and KL152, respectively. Moreover, the wzi allele of KP-JZ107 was defined as wzi 209.

### 3.6. Virulence-Associated Genes

A large number of virulence-associated genes among KP-JZ107 and AB-JZ67 isolates were identified, including *rmpA/A2* and *rscAB* (virulence regulation genes), *iucA, iucB, iucC, iucD,* and *iutA* (aerobactin), HPI (yersiniabactin), colibactin, type 1 and type 3 fimbriae and siderophores (*entA, entB, entC, entD, entE, entF, entS, febA, febB, febC, febD,* and *febF*), salmochelin (*iroC, iroD, iroE,* and *iroN*), lipopolysaccharide biosynthetic locus (*rfb*), type VI secretion system, and all ABCDRS (allantoin utilization), which were identified using VirulenceFinder 2.0 (Table 2).

In KP-JZ107 genome, we identified the main types of fimbriae including type 1 (*fimA, fimB, fimC, fimD, fimE, fimF, finG, fimH, fimI,* and *fimK* genes) and type 3 (*mrkA, mrkB, mrkC, mrkD, mrkF, mrkH, mrkI,* and *mrkJ* genes) fimbriae. The gene *rmpA* is related to regulation of the synthesis of capsular polysaccharides and was also found in the KP-JZ107 isolate. KP-JZ107 also contained genes related to major iron-uptake molecules, including enterobactin (*entA, entB, entC, entD, entE, entF, entS, febA, febB, febC, febD,* and *febF* genes), aerobactin (*iucA, iucB, iucC,* and *iucD* genes), yersiniabactin (*ybtA, ybtE, ybtP, ybtQ, ybtS, ybtT, ybtU,* and *ybtX* genes), and salmochelin (*iroB, iroC, iroD, iroE,* and *iroN,* genes). Additionally, KP-JZ107 presented polyketide synthase genes (*clbA, clbB, clbC, clbD, clbE, clbF, clbG, clbH, clbI, clbJ, clbL, clbM, clbN, clbO, clbP, clbQ,* and *clbS*), which are responsible for the synthesis of bacterial colibactin (Table 2 and Figure 1).

Genome analysis of the AB-JZ67 isolate revealed that genes involved in the formation of biofilm (*bap* and *pgaA, pgaB, pgaC,* and *pgaD*) and pili (*csu*) were detected. We also identified genes associated with adherence (*ompA*), serum resistance (*pbpG*), oxidative stress (*katA*), synthesis of lipid A (*lpxA, lpxB, lpxC, lpxD, lpxL,* and *lpxM*), and LPS core (*lpsB*) in the isolates. Moreover, the AB-JZ67 isolate showed the iron-uptake gene (*hemO)*, acinetobactin genes (*bau, bas, bar,* and *ent*), and regulators of quorum sensing (*aba*) and the component system (*bfm*) (Table 2 and Figure 1).

### 3.7. Antimicrobial and Heavy Metal-Resistant Genes

The presence of ARGs in the KP-JZ107 and AB-JZ67 genomes was characterized using ResFinder 4.1, and the results are shown in Table 3 and Figure 1. KP-JZ107 harbored *β*-lactam genes *bla*_SHV−12_, *bla*_TEM−1B_, *bla*_CTX−M−65_, *bla*_NDM−1_, and *bla*_KPC−2_, while AB-JZ67 carried the *bla*_ADC−25_, *bla*_OXA−23_, and *bla*_OXA−66_ genes. The resistance to aminoglycosides (amikacin and gentamicin) was mainly mediated by *armA* and *rmtB* in the KP-JZ107 isolate and by *aph*(3′)-VI in the AB-JZ67 isolate. Resistance to tetracycline was acquired by mutated *ramR* in KP-JZ107 and via *tet*(B) in AB-JZ67. Both isolates harbored *msr*(E) and *mph*(E) genes, which confer resistance to macrolides. The *sul2* gene encoding sulfonamide resistance was found in both isolates, whereas *sul1* was detected in only KP-JZ107. Other resistance genes were also detected in KP-JZ107, including *oqx*A/B (quinolone and amphenicol resistance), *fos*A and *fos*A3 (fosfomycin resistance), and *cat*A2 (chloramphenicol resistance). The KP-JZ107 isolate exhibited specific mutations at the loci S80I in parC, D350N, and S357N in PBP3, D87G in gyrA, and E350Q in UhpT. To further investigate, ResFinder V.4.6 was employed to identify AMR genes with the highest number of mutations. Analysis results revealed several mutations (*n* = 6) in the *ramR* gene and seven mutant sites in the *acrA* gene, both of which encode components of the multidrug efflux pump. The highest number of mutations were observed in the genes encoding the Ompk35 (*n* = 14) and Ompk37 (*n* = 12) porin proteins. These mutations in the pore proteins OmpK35 and OmpK37 contribute to the obstruction of carbapenem drugs from entering the bacterial cells. The AB-JZ67 isolate showed mutations at the S81L locus in *gyrA*, and V104I and D105E in *parC*, which confer high-level resistance to fluoroquinolones.

### 3.8. Mobile Genetic Elements (MGEs)

Various MGEs were identified in our isolates using PlasmidFinder and MobileElementFinder database. Five plasmid replicons were detected in only KP-JZ107 (IncFII [pHN7A8], IncFIB[pNDM-Mar], IncHI1B [pNDM-Mar], IncR and CoIRNAI), while different insertion sequences (ISs) were found in both isolates. KP-JZ107 showed the following ISs: IS102, IS6100, ISCfr1, ISVsa3, ISEc9, ISEc29, ISKpn1, ISKpn14, ISKpn18, ISKpn19, ISKpn21, ISKpn27, and ISKpn28. Four ISs (ISAba10, ISAba26, IS26, and ISVsa3) and only one transposon (Tn6022) were detected in the AB-JZ67 isolate.

### 3.9. Phylogenetic Analysis

**Figure 2:**
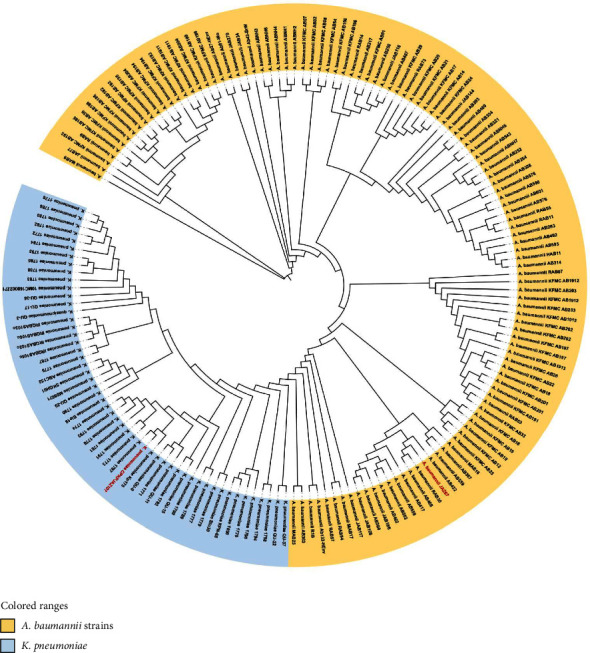
A phylogenetic tree based on whole-genome sequencing (WGS) data of the current isolates (KP-JZ107 and AC-JZ67) with specific strains (22 *K. pneumoniae* and 105 *A. baumannii*) from Gulf Cooperation Council (GCC) countries, which were previously identified in the BV-BRC database, was obtained using the BV-BRC Bacterial Genome Tree Service. The interactive tree of life tool (iTOL) was used to visualize the phylogenetic tree. Red isolates: the current isolates.

We constructed the phylogenetic relationship between KP-JZ107 and AC-JZ67 with specific strains (22 *K. pneumoniae* and 105 *A. baumannii* strains) from countries in the Gulf Cooperation Council (GCC) using previously identified strains in the BV-BRC database. This analysis was performed using the BV-BRC Bacterial Genome Tree Service (Figure 2). The phylogenetic tree showed that *K. pneumoniae* KP-JZ107 was phylogenetically clustered in a single clade with ST11 strains (KP1797 and KP175) that were isolated from Oman (GenBank JAURSG000000000) and UAE (GenBank JAVCPJ000000000), respectively (Figure 2). In contrast to KP-JZ107, KP1797 and KP175 carry only a single carbapenemase gene.

*A. baumannii* AB-JZ67 was classified into a distinct cluster alongside three local strains (AB466 LYNQ00000000, AB432 LYNP00000000, and RAB30 CP121581), which exhibited the highest degree of phylogenetic relatedness based on their genetic structure and ST2 (Figure 2). Notably, AB466 and RAB30 shared the same resistance genes (*bla*_*OXA*−66_, *bla*_*OXA*−23_, and *tet*(B)) with our isolate.

## 4. Discussion

In this study, we characterized NDM-1 and KPC-2 coproducing *K. pneumoniae* (KP-JZ107) and OXA-23 and OXA-66-producing *A. baumannii* (AB-JZ67) isolates that were recovered from a BSI in a critical ill patient with COVID-19 in southwestern Saudi Arabia. To the best of our knowledge, this is the first genomic analysis of bacterial coinfection due to tigecycline/carbapenem-resistant *K. pneumoniae* and *A. baumannii* in Saudi Arabia, and their resistance antibiograms have indicated that the isolates were XDR. Since the initial report in the central region of Saudi Arabia, we also described a *bla*_*KPC*−2_-harboring *K. pneumoniae* isolate in southern Saudi Arabia for the first time [11].

KP-JZ107 was designated as ST11 and belonged to clonal complex 258 (CC258). It is considered a global public health issue due to its broad global distribution and AMR. In addition, ST11 has recently been associated with *bla*_*NDM*−1_ and *bla*_*KPC*−2_ coproducing *K. pneumoniae* strains in many studies [12, 13]. The KP-JZ107 isolate was characterized as the KL47 (wzi-209) capsular serotype. Consistent with this observation, previous studies have demonstrated that the ST11 KL47 clone represents a high-risk lineage and is the predominant serotype in carbapenem-resistant *K. pneumoniae* (CRKP) infections, which have been implicated in numerous nosocomial outbreaks across Asia [14–16]. We propose that the transmission of ST11-KL47 (wzi-209)-associated BSIs is prevalent in Asia, and its unusual emergence in Saudi Arabia requires more attention. To date, we have not documented any other *K. pneumoniae* isolates in other Saudi regions that exhibit the same resistance mechanism and ST as KP-JZ107. Unfortunately, our patient passed away, preventing us from obtaining further information about colonization.

There was a notable difference from the *bla*_NDM−1_ and *bla*_KPC−2_ coproducing ST11 isolates previously reported in China in that the current isolate KP-JZ107 exhibited resistance to tigecycline [12]. Although the mechanisms of tigecycline resistance are not yet fully understood, the most commonly reported mechanisms include overexpression of genes encoding the resistance-nodulation—cell division family (RND) efflux pumps AcrAB (such as *ramA* and *ramR*), acquisition of *tet* (A) variants, and ribosomal protein change (*rpsJ*) genes [17]. We observed that the *acrAB* genes and the *ramR* mutation both contributed to a slight increase in the minimum inhibitory concentration (MIC) to > 4 mg/L, which conferred an adequate level of tigecycline resistance in *K. pneumoniae* KP-JZ107. Most aminoglycoside-resistant mechanisms are associated with phosphotransferases (APHs) and 16S rRNA methyltransferase (16S RMTase)-encoding genes [18]. In the present study, the simultaneous presence of *rmtB* and *armA* in KP-JZ107 was consistent with prior isolates from China, demonstrating a high level of resistance to aminoglycosides [19]. Tigecycline and aminoglycosides are commonly utilized for the treatment of CRKP infections. However, the findings require careful therapeutic approaches, and it is crucial to highlight the developing resistance of CRKP to these drugs, or else an epidemic of MDR may pose a fatal threat. Mutations in the Ompk35, Ompk36, and OmpK37 porin proteins play a critical role in MDR in CRKP. These mutations can lead to structural changes that reduce the permeability of the bacterial outer membrane and limit the entry of antibiotics, particularly beta-lactams and carbapenems [20]. In the current study, the highest number of mutations was observed in Ompk35 and OmpK37 of KP-JZ107.

Capsular serotypes KL1 and KL2 are typical characteristics of hypervirulent *K. pneumoniae* (hvKP) strains. KL47 can also cause serious infections and exhibits high virulence. However, hypercapsule production is not necessarily associated with hypermucoviscosity [21]. Thus, the capsular serotype may be associated with bacterial virulence or can enhance virulence, but it is not the only virulence-associated marker of hvKP strains. The regulation of capsule synthesis is mainly mediated by *rmpA* and *rmpA2*, which promote high mucoviscosity phenotype of hvKP strains [22]. Moreover, *K. pneumoniae* virulence plasmids (KpVPs), particularly KpVP-1 (harboring *iuc*1, *iro*1, *rmp*A, and *rmp*A2), have commonly been associated with hvKP strains [23].

Taken together, our findings suggest that the hypervirulent phenotype of KP-JZ107 is likely associated with increased mucoviscosity, which may be mediated by the presence of capsule synthesis regulators and the KpVP-1 plasmid. However, the precise mechanism underlying this association warrants further investigation. Plasmids are the major vehicles that are responsible for the dissemination of AMR and are often associated with bacterial pathogenicity. Their duplication and survival capacity depend on their replication determinants (replicons) [24]. The genome of *K. pneumoniae* continuously acquires AMR genes via the incorporation of plasmids and other MGEs, such as transposons (Tns) and integrons. This genetic exchange leads to the emergence of strains exhibiting MDR and XDR, which poses significant challenges for effective treatment options. The incompatibility group F (IncF) plasmids have commonly been identified among ST11/KPC-2 strains particularly in China [25]. The clonal dissemination of these strains is also associated with conjugative plasmids, including ColRNAI, IncFII(pHN7A8), and IncR [26], which were detected in our KP-JZ107 isolate. Consistent with our findings, the plasmids IncFII, IncFIB, and IncHI1B were also detected in the ST11/NDM hvKP isolate, which was recovered from a patient with bacteremia in India [27]. Remarkably, our (KP-JZ107) isolate harbored IncHI1B (pNDM-Mar), which was first described as a *bla*_NDM_-harboring plasmid in *K. pneumoniae* [28]. Moreover, the virulence of *K. pneumoniae* is highly associated with KpVPs that are typically large IncFIBk replicons. In the present study, we found KpVP-type-1, which mainly carries *iuc2, iro1, rmpA,* and *rmpA2* [29]. The emergence of this isolate, which harbors plasmid-mediated AMR and virulence genes, reflects the genomic evolution of these superbugs and could constitute a potential threat to healthcare facilities. The genomic profiles of two local *K. pneumoniae* strains KP1797 and KP175 exhibit a high degree of similarity to our isolated strain, which supports the association of ST11 with the presence of *bla*_*KPC*−2_ and/or *bla*_*NDM*−1_ resistance genes. Despite the isolation of KP175 in 2012, the dissemination of ST11 across the region including Saudi Arabia appears to be sporadic. This distribution pattern is not an unexpected occurrence for other ST11 strains, which are recognized as globally predominant and associated with a high risk [30]. Therefore, ongoing epidemiological surveillance of this clone is crucial for effective public health management and disease prevention strategies.

The genomic analysis of MDR *A. baumannii* is crucial for understanding its transmission dynamics within ICUs that receive critically ill patients with COVID-19. Current research indicates that ST2 *A. baumannii* is the most prevalent and widely distributed clone in many regions worldwide, including Saudi Arabia [31]. Recently, Cherubini et al. reported on the prevalence of ST2 strains exhibiting significant resistance to last-resort antimicrobials, which were isolated from severely ill patients with COVID-19 and BSIs in the ICU [32]. Remarkably, the *bla*_*OXA*−23_ and *bla*_*OXA*−66_-coproducing *A. baumannii* ST2 clone is a high-risk clone that is prevalent worldwide, disseminating internationally, and represents a highly potential risk to global public health [33]. The increased AMR in *A. baumannii* is largely due to the dynamics of MGEs, the activation of intrinsic resistance mechanisms such as the chromosomal *β*-lactamase *bla*_*ADC*_, and the presence of efflux pumps [34].

In this study, we found the presence of *bla*_*ADC*−25_ cephalosporinase and adeFGH RND efflux pumps in our ST2 AB-JZ67 isolate. To the best of our knowledge, these efflux pumps appear to play a major role in conferring resistance to various antibiotic classes in *A. baumannii* [35]. Furthermore, ST2 strains are strongly associated with the resistance to *β*-lactam agents with the coexistence of *bla*_*ADC*−25_, *bla*_*OXA*−23_, and *bla*_*OXA*−66_ [36]. Consequently, the prevalent coexistence of *bla*_*OXA*−23_ and *bla*_*OXA*−66_, along with several other carbapenem-hydrolyzing enzymes in *A. baumannii*, offers a scientific explanation for the reduced efficacy of *β*-lactam drugs in clinical settings.

In addition to the presence of *β*-lactam–resistant genes, the local AB-JZ67 isolate demonstrated an XDR pattern. This resistance was observed against multiple antimicrobial agents, as evidenced by both phenotypic testing and genomic identification of various resistance mechanisms. Aminoglycoside resistance in *A. baumannii* is predominantly facilitated by the presence of specific resistance genes, including *armA, aph(6)-Id,* and *aph(3″)-Ib*, which contribute to the reduced susceptibility of these bacteria to aminoglycosides [37]. The *aph(3′)-VIa* gene has primarily been described in *A. baumannii,* and its activity confers resistance to amikacin and kanamycin rather than gentamycin [38]. In the ST2 AB-JZ67 isolate, we observed resistance genes *aph(3″)-Ib, aph(3′)-VIa,* and *aph(6)-Id*, which are associated with aminoglycoside resistance. Additionally, *A. baumannii* demonstrated reduced sensitivity to various antibacterial agents, such as tigecycline and tetracycline. This decreased susceptibility can be attributed to the overexpression of efflux pump genes *adeF, ade, adeG, adeH,* and *tet*(B) [39]. Accordingly, these mechanisms could be responsible for tigecycline resistance in the current isolate AB-JZ67. Simultaneously, the AB-JZ67 isolate exhibited mutations at the S81L locus in *gyrA*, as well as V104I and D105E in parC, which confer high-level resistance to fluoroquinolones. These mutations were found to be prevalent in MDRAB, particularly within ST2 clones in many countries [40].

Consequently, clinical isolates of *A. baumannii* frequently exhibit multidrug-resistant phenotypes, which enables their prolonged survival in healthcare environments and reduces the available therapeutic options. The AB-JZ67 isolate harbors an array of genes associated with major classes of virulence factors, including adherence factors, biofilm formation, immune system evasion, iron uptake, regulation, and serum resistance. The presence of these virulence determinants could potentially increase the pathogenicity and exacerbate the severity of infections caused by this particular isolate [36]. The formation of biofilms in *A. baumannii* has been hypothesized to impede the diffusion of antibiotics across bacterial cells, thereby contributing to the development of MDR phenotype. This biofilm formation also enhances the robust survival capabilities of *A. baumannii* in harsh environmental conditions [41]. The abaI/R quorum sensing system was detected in the AB-JZ67 isolate and is largely involved in motility, growth features, and biofilm formation [42]. In addition, biofilm-associated protein (BAP) enhances the formation of complex *A. baumannii* community structures on abiotic surfaces, including invasive medical devices, by promoting biofilm development and stability [43]. Accordingly, the formation and persistence of bacterial biofilm communities within hospital settings, particularly in ICUs, could potentially allow biofilms to act as substantial reservoirs of infections for immunocompromised patients.

Regarding MGEs in AB-JZ67, genomic resistance island AbaR (“A*. baumannii* Resistance”) are well-known elements that can cause multiple AMRs in *A. baumannii*. These elements display variable genetic structural features, involving certain different but closely related molecular backbones and acquired transposable elements such as Tns, ISs, and AMR genes based on genetic elements [44]. The *bla*_*OXA*−23_ gene, for example, is predominantly associated with the transposon Tn2006. Due to its transposition capability, Tn2006 undergoes structural rearrangement resulting in the formation of Tn6022, which is also known as AbaR4. This rearrangement process seems to play a significant role in facilitating the dissemination of *bla*_*OXA*−23_ genes [45].

A comparative analysis revealed that the genetic composition of the AC-JZ67 isolate had a high degree of similarity with two local strains AB466 and RAB30 and had identical primary resistance mechanisms. AB466 was initially isolated in 2013, and there has been a notable increase in the prevalence of *A. baumannii* ST2 clones coproducing OXA-23 and OXA-66 over the past decade. This pattern suggests the persistent circulation of the ST2 strains within healthcare facilities in Saudi Arabia.

In conclusion, AMR poses a significant global challenge for healthcare institutions, particularly in ICUs. The COVID-19 pandemic has further exacerbated the complexity and prevalence of MDR infections, especially among patients requiring mechanical ventilation. This study represents the first documented case of a coinfection with two highly antibiotic-resistant isolates in a patient with COVID-19 in the southern region of Saudi Arabia. We aimed to gain insights into the epidemiological behavior and identify the predominance and endemicity patterns within our healthcare facilities. Our findings suggest that the coexistence of these bacteria may indicate their widespread presence in ICUs, requiring comprehensive surveillance studies across all hospitals. Identifying both endemic and epidemic patterns is crucial for implementing effective control measures and reducing the prevalence of antibiotic-resistant infections in healthcare settings.

## Figures and Tables

**Table 1:** Antibiotic susceptibility profile of *K. pneumoniae* (KP-JZ107) and *A. baumannii* (AB-JZ67) isolates recovered from blood culture of critically ill COVID-19 patient.

Antimicrobial agents	KP-JZ107		AB-JZ67	
	MIC (mg/L)	Interpretation	MIC (mg/L)	Interpretation
Amikacin	> 32	R	> 32	R
Amoxicillin/clavulanic acid	> 16/8	R	> 16/8	R
Ampicillin	> 16	R	> 16	R
Aztreonam	> 16	R	16	R
Cefepime	> 16	R	> 16	R
Cefoxitin	> 16	R	> 16	R
Ceftazidime	> 16	R	> 16	R
Ceftriaxone	> 32	R	> 32	R
Cefuroxime	> 16	R	> 16	R
Cephalothin	> 16	R	> 16	R
Ciprofloxacin	> 2	R	> 2	R
Colistin	≤ 1	S	≤1	S
Ertapenem	> 4	R	> 4	R
Gentamicin	> 8	R	> 8	R
Imipenem	> 8	R	> 8	R
Meropenem	> 8	R	> 8	R
Levofloxacin	> 4	R	> 4	R
Piperacillin/tazobactam	> 64/4	R	> 64/4	R
Tigecycline	> 4	R	> 4	R
Nitrofurantoin	> 64	R	> 64	R
Trimethoprim/sulfamethoxazole	> 4/76	R	> 4/76	R

**Table 2:** Virulence factors of *K. pneumoniae* ST11 (KP-JZ107) and *A. baumannii* ST2 (AB-JZ67) recovered from bloodstream infection in critical ill COVID-19 patient in southwestern Saudi Arabia, based on the virulence factor database.

Category	Virulence factors	Related genes	
		KP-JZ107	AB-JZ67
Adherence	Type-1 fimbriae	*fimA, fimB, fimC, fimD, fimE, finF, fimG, fimH, fimI, and fimK*	
	Type-3 fimbriae	*mrkA, mrkB, mrkC, mrkD, mrkF, mrkH, mrkI, and mrkJ*	
	Type IV pili	*pilW*	
	Outer membrane protein		*ompA*

Biofilm formation	AdeFGH efflux pump/transport autoinducer		*adeF, adeG, and adeH*
	Biofilm-associated protein		*Bap*
	Csu pili		*csuA/B, csuA, csuB, csuC, csuD, and csuE*
	PNAG (polysaccharide poly-N-acetylglucosamine)		*pgaA, pgaB, pgaC, and pgaD*

Efflux pump	AcrAB	*acrA and acrB*	

Enzyme	Phospholipase C		*plcC*
	Phospholipase D		*plcD*

Immune evasion	LPS		*lpxA, lpxB, lpxC, lpxD, lpxL, lpxM, and lpsB*

Iron uptake	Aerobactin	*iucA, iucB, iucC, iucD, and iutA*	
	Enterobactin	*entA, entB, entC, entD, entE, entF, entS, febA, febB, febC, febD, febF, and fes*	
	Salmochelin	*iroB, iroC, iroD, iroE, and iroN,*	
	Yersiniabactin	*fyuA, irp1, irp2, ybtA, ybtE, ybtP, ybtQ, ybtS, ybtT, ybtU, and ybtX*	
	Acinetobactin		*barAB, basA, basB, basC, basD, basF, basG, basH, basI, basJ, bauA, bauB, bauC, bauD, bauE, bauF, and entE*
	Heme utilization		*hemO*

Nutritional factor	Allantoin utilization	*allA, allB, allC, allD, allR, and allS*	

Regulation	RcsAB	*rcsA and rcsB*	
	RmpA	*rmpA*	
	Quorum sensing		*abaI and abaR*
	Two-component system		*bfmR and bfmS*

Secretion system	T6SS-I	*clpV/tssH, dotU/tssL, hcp/tssD, icmF/tssM, impA/tssA, ompA, sciN/tssJ, tle1, tli1, tssF, tssG, vasE/tssK, vgrG/tssI, vipA/tssB, and vipB/tssC*	
	T6SS-II	*clpV, dotU, icmF, impF, impH, impJ, ompA, sciN, vasA/impG, and vgrG*	
	T6SS-III	*dotU, icmF, impA, impF, impG, impH, impJ, lysM, ompA, sciN, and vgrG*	

Serum resistance	PbpG		*pbpG*

Toxin	Colibactin	*clbA, clbB, clbC, clbD, clbE, clbF, clbG, clbH, clbI, clbJ, clbL, clbM, clbN, clbO, clbP, clbQ, and clbS*	

Stress adaptation	Catalase		*katA*

**Table 3:** Antimicrobial-resistant genes that were detected in *K. pneumoniae* (KP-JZ107) ST11 and *A. baumannii* ST2 (AB-JZ67) isolated from bloodstream infection in critically ill COVID-19 patient in southwestern Saudi Arabia.

Class	Antibiotic	Related genes	
		KP-JZ107	AB-JZ67
*β*-Lactam	Ampicillin, amoxicillin/clavulanic acid, piperacillin/tazobactam, cefoxitin, cefepime, ceftazidime, ceftriaxone, cefotaxime, imipenem, meropenem, ertapenem, and aztreonam	*bla*_*SHV*−12_, *bla*_*TEM*−1*B*_, *bla*_*NDM*−1_, *bla*_*KPC*−2_, and *bla*_*CTX*−*M*−6_5	*bla*_*ADC*−25_, *bla*_*OXA*−23_, and *bla*_*OXA*−66_
Aminoglycoside	Amikacin and gentamicin	*rmtB, armA, and aph(3')-VI (amikacin)*	*aph(3”)-Ib, aph(3')-VIa, and aph(6)-Id*
Macrolide	Azithromycin and erythromycin	*msr(E), mph(A), and mph(E)*	*msr(E) and mph(E)*
Quinolone	Ciprofloxacin and levofloxacin	*oqxB, oqxA, qnrS1, parC, gyrA, and gyrB*	
Amphenicol	Chloramphenicol	*oqxA, oqxB, and catA2*	
Fosfomycin	Fosfomycin	*fosA3 and fosA*	
Tetracycline	Tigecycline	*ramR*	*tet(B)*
Folate pathway antagonist	Trimethoprim/sulfamethoxazole	*Sul1, sul2, oqxA, and oqxB*	*sul2*

## Data Availability

Whole genome sequence data presented in this article have been uploaded onto the NCBI under the BioProject ID PRJNA110342 (will be released upon acceptance). Other data utilized to support the findings of this research can be obtained from the corresponding author upon request (iaalzahrani1@kau.edu.sa).
